# Progress in Ophthalmology

**Published:** 1896-11-14

**Authors:** 


### Progress in Ophthalmology.

The treatment of trachoma is still a fruitful subject in the journals. Strouse (*Therapeutic Gazette*, May 15th, 1896) strongly recommends curetting by means of a sharp spoon of 1·5 millimetres in width. Each lid is everted in turn after the application of cocaine, and the whole conjunctival surface involved is thoroughly scraped. When all bleeding has ceased the surfaces are washed with either boracic acid or perchloride of mercury solutions, then touched up with a 2 per cent. solution of silver nitrate. The process is attended with considerable reaction, which is allayed by iced pads. The application of the caustic solution is made each alternate day for eight or ten days; after that it may be varied by using sulphate of copper. Strouse holds that by this method there is much less cicatricial tissue formed than by "grattage" or scarification, so that the mucous surface is restored almost in its entirety, and that the cure is almost invariably a radical one.

Stephenson (*Brit. Med. Journal*, July 11th, 1896) records an outbreak of mucopurulent ophthalmia in the Central London District School, and demonstrates how prompt isolation may quickly rid a school community of such a danger. Between April 12th and April 25th twenty-five children became affected, and of these twenty-two came from one ward and the other three from dormitories close by. The success of the treatment is the more pronounced when we find in no case was there any corneal involvement or any sequelæ. The writer of this article had occasion last year to deal with an outbreak in an industrial school whose habitation was an old hulk; the hygienic conditions were such that there was great danger of the trouble becoming endemic. Whilst the school-room was a well-aired structure erected on the deck, the dormitories were the ordinary 'tween-decks of an old man-of-war, with barely standing room. The boys used towels which were common to squads. Fortunately, there was at one end of the ship, and completely isolated from the rest, a well-appointed hospital ward, and by strict isolation and rigid inspection of the whole ship's company daily, the progress was in a short time arrested, and there were no bad results.

Dr. W. H. Bates (*New York Medical Record*, March 28th, 1896) advocates the use of a glass syringe in all cases of acute or chronic lachrymal disease, and deprecates the use of astringents and antiseptics. He maintains that better effects can be obtained from the use of water, the syringing being kept up till clear fluid comes away. The process must be a somewhat trying one, as he says that in some cases as much as a pint must be so used at a sitting; as further he says it must be done daily, and that chronic cases required it to be continued for months, it hardly seems a practical suggestion, as few patients would have the patience to undergo the treatment.

In France there are some 38,000 to 39,000 blind persons, and it has been estimated that from 30 to 40 per cent. of these were due to ophthalmia neonatorum. The subject was under discussion at the French Society of Ophthalmology (*The Medical Week*, May 8th, 1896), and Dr. Hone, of Buffalo, advocated the putting in force in France of some such stringent law as holds good in the United States, and affects twenty millions of inhabitants. Amongst its provisions we note the following:—

"1. If a midwife or nurse having charge of an infant finds that one or both eyes of said infant are red or inflamed at any time during the two weeks immediately following birth, the midwife or nurse is to report the fact in writing within six hours to the health officer or any legally qualified practitioner of medicine in the city, town, or district in which the parents of the child reside.

"2. Each infraction of the law is punishable by a fine not to exceed 500 francs, imprisonment during a period not to exceed six months, or both fine and imprisonment."

Dr. Hone's proposal did not meet with full approval from those present, some considering that it would be looked upon as an interference with personal liberty, but the compulsory notification was considered to be most wise. It is said to have acted most beneficially in Switzerland, where it has been in force since 1865.

A writer (*Journal des Pratiques*, Jan., 1896) points out that there is a great tendency to recurrence in styes largely due to auto-inoculation. Great care should be taken, therefore, that the edges of the lids shall be kept thoroughly cleansed with some antiseptic both at the time of the occurrence of a stye and for some time after.

Chibret (*Société d'Ophthalmol de Paris*, Feb., 1896) recommends an alcoholic solution of methyl-violet in the treatment of infected corneal ulcers. He applies a 10 per cent. solution on a probe enwrapped with cotton wool directly to the ulcerated part daily. The stain which it imparts to the tissues lasts a considerable time —twenty-four or more. The application causes no great pain. He deprecates the use of the cautery as unnecessarily increasing the area of scar tissue. He also recommends instillation of solution of cyanide of mercury hourly. If hypopion is present he withdraws the pus by opening the anterior chamber.

Van der Bergh (*Indian Med. Chir. J.*, April, 1896) strongly advocates treatment of these cases with tincture of iodine, applied in the same manner as Chibret uses the methyl-violet, twice daily. He uses afterwards atropine and a compress bandage.

Many operators have attempted to graft portions of clear cornea on to the human eye, but so far with no practical success; the corneal graft may live, but sooner or later it has always become opaque. It was first attempted by Rieke (*American Med. Surg. Bulletin*, April 18th, 1896) in 1823. Thirty years later Nussbaum tried to insert an artificial cornea of glass, which fitted like a stud in the opaque cornea; it gave only temporary vision, and speedily started inflammatory mischief. Wolfe, Hippel, Argyll Robertson, among others, have all succeeded in getting grafts to live, but only to become subsequently opaque.

Suker (*Toledo Med. Rept.*, 1895) has experimented on the lower animals, but, although readily getting union and maintained vitality in his grafts, there was no permanent transparency. It would be of inestimable benefit if some method of keratoplasty could be devised.

For pterygium Deschamps (*Med. Chronicle*, May, 1896) recommends curetting of the cornea over the area from which the pterygium has been detached. He says it is necessary to remove all the deeper attachments to the cornea, and that this does it as effectually as the thermo-cautery, and without leaving a scar.

Simple chronic glaucoma is probably one of the most disappointing of all eye diseases to deal with, but the general opinion amongst ophthalmologists seems now against operative procedure. The name itself is, perhaps, a misleading one, and it might be better to adopt the suggestion of Knies (*Arch. of Ophthal*, XXIV., 2), and call these "cases of optic nerve atrophy with excavation." Abadie (*New York Med. Journal*, April, 1896) deprecates all operative interference, and advocates systematic and prolonged use of miotics. He uses eresene and pilocarpine, or both, daily, and also gives fifteen to thirty grain doses of bromide of potassium, and on alternate days, quinine. This is continued for a month, and then suspended for eight days and resumed.

Another suggested treatment, galvanism, finds an advocate in Pilgrim (*Ann. of Ophth. and Otol.*, IV., 2). As he uses pilocarpine at the same time it is, as he admits, doubtful as to what extent the galvanism is to be credited with the favourable result. He considers that a catalytic action is set up, by means of which the products of malnutrition are destroyed, and restoration of function takes place by the changing of the perverted to a healthy nutrition.

An extremely interesting discussion took place at the meeting of the French Society of Ophthalmolology (*Medical Week*, May 15th, 1896) in May last, on "Extraction of the Crystalline Lens as a Prophylactic Measure against High Progressive Myopia and detachment of the Retina." The subject was introduced by Dr. Vachar, who pointed out that the originator of this procedure was the Abbé Desmonceaux, who used it in 1776. Referring to an objection raised at a former discussion that a patient, myopic to the extent of 25 or 30 dioptres, is still highly myopic after removal of the lens, he pointed out that, as a result of experience, he agreed with the observations of Eperon, that after this operation a myope of 20 dioptres becomes emmetropic, and one of 30 dioptres has a reduction to three dioptres. He urged that the operation should be performed when there is rapidly progressing myopia between the ages of twelve and sixteen. It may be done any time after the age of three, if there is staphyloma, and the number of dioptres of myopia exceeds that of the patient's years. Only one eye should be operated on, preferably the one more seriously affected; the other might be subsequently operated on if the myopia continued progressive.

After 30 years of age a myope of over 15 dioptres is peculiarly liable to detachment of retina, and in these cases it should unhesitatingly be performed.

Dr. Abadie said that the indication for the operation is commencing macular choroido-retinitis or other fundal changes.

Chibret spoke of excellent results obtained in high myopia. He performs discission with a broad cystotome and extracts by means of a syringe four or five days later. Galezowski thought the operation justifiable only when the myopia exceeds fifteen dioptres. Both he and Pflüger agreed that macular lesions were an indication for operating.

